# Perceived barriers and opportunities to improve working conditions and staff retention in emergency departments: a qualitative study

**DOI:** 10.1136/emermed-2023-213189

**Published:** 2024-01-09

**Authors:** Jo Daniels, Emilia Robinson, Elizabeth Jenkinson, Edward Carlton

**Affiliations:** 1 Department of Psychology, University of Bath, Bath, UK; 2 Psychology, North Bristol NHS Trust, Westbury on Trym, Bristol, UK; 3 Department of Health and Social Sciences, University of the West of England, Bristol, UK; 4 Emergency Department, Southmead Hospital, North Bristol NHS Trust, Westbury on Trym, UK; 5 Bristol Medical School, University of Bristol, Bristol, UK

**Keywords:** qualitative research, staff support

## Abstract

**Background:**

Staff retention in Emergency Medicine (EM) is at crisis level and could be attributed in some part to adverse working conditions. This study aimed to better understand current concerns relating to working conditions and working practices in Emergency Departments (EDs).

**Methods:**

A qualitative approach was taken, using focus groups with ED staff (doctors, nurses, advanced care practitioners) of all grades, seniority and professional backgrounds from across the UK. Snowball recruitment was undertaken using social media and Royal College of Emergency Medicine communication channels. Focus group interviews were conducted online and organised by profession. A semi-structured topic guide was used to explore difficulties in the work environment, impact of these difficulties, barriers and priorities for change. Data were analysed using a directive content analysis to identify common themes.

**Results:**

Of the 116 clinical staff who completed the eligibility and consent forms, 46 met criteria and consented, of those, 33 participants took part. Participants were predominantly white British (85%), females (73%) and doctors (61%). Four key themes were generated: ‘culture of blame and negativity’, ‘untenable working environments’, ‘compromised leadership’ and ‘striving for support’. Data pertaining to barriers and opportunities for change were identified as sub-themes. In particular, strong leadership emerged as a key driver of change across all aspects of working practices.

**Conclusion:**

This study identified four key themes related to workplace concerns and their associated barriers and opportunities for change. Culture, working environment and need for support echoed current narratives across healthcare settings. Leadership emerged more prominently than in prior studies as both a barrier and opportunity for well-being and retention in the EM workplace. Further work is needed to develop leadership skills early on in clinical training, ensure protected time to deliver the role, ongoing opportunities to refine leadership skills and a clear pathway to address higher levels of management.

**WHAT IS ALREADY KNOWN ON THIS TOPIC**

- Retention of staff in emergency medicine is at crisis level and has been a high priority area for over a decade.
- Multiple guidelines have been published to outline improvements that need to be made to retain staff; however, little improvement has been seen on the ground in EDs.
- Key factors such as staff burnout and poor working conditions are known to influence intention to leave; however, it is unclear why change has not taken place despite knowledge of these problems and existing guidelines seeking to address these issues.

**WHAT THIS STUDY ADDS**

- This qualitative study assessed perceived barriers that may be inhibiting the implementation to working conditions and working practices in EDs.
- Leadership is identified as an important driver of change in working practices and can play an important role in workplace well-being and retention.
- Key recommendations for avenues of improvement are made, identifying key actions at government, professional, organisational and personal level.

**HOW THIS STUDY MIGHT AFFECT RESEARCH, PRACTICE OR POLICY**

- This study identifies leadership as a key opportunity for change and as a result makes specific recommendations for policy and practice regarding leadership in emergency medicine.

## Introduction

Emergency Medicine (EM) is facing a global staffing crisis.^1^ Record numbers of staff continue to leave the UK NHS with EM the most affected specialty.^2^ EM reports the highest work intensity of all medical specialties,^3^ with ‘intensity’ recognised as one of the leading factors in job dissatisfaction, attrition and career burnout.^3–5^ These factors are amplified in an already stretched workforce.^2^ Psychological well-being of the EM workforce is compromised, with working conditions recognised as playing a key role.^6 7^ Staff attrition has a systemic impact: lower staff ratios lead to higher workloads, reduced quality of care,^8^ higher levels of medical errors^9^ and poorer staff well-being,^10^ all factors associated with staff absence and intention to leave.^11^ The landscape of EM has also changed; increased prevalence of high patient acuity, multimorbidity and an ageing population all bear considerable impact.

Key sector stakeholder initiatives and policy recommendations relating to retention and well-being^12–14^ are largely generic and forfeit relevance to the specialty due to the lack of specificity to the clinical context within which these guidelines need to be implemented. Retention improvement programmes suggest approaches should be tailored per organisation,^12^ however, this assumes that the challenges faced by staff across specialities and disciplines are homogeneous. In a specialty which reports the highest pressured environment, highest attrition and rates of burnout,^15^ considerations of workplace context and specificity of policy recommendations are likely to be crucial. Interventions or initiatives must take account of the unique demands of the EM working environment, and how feasible it is to implement recommendations.

The James Lind Alliance (JLA) priority setting partnership in EM^16^ identified initiatives to improve staff retention as research priorities in 2017 and again in the 2022 JLA refresh,^17^ signalling the need for further research in this area due to a deepening workforce crisis. Current guidelines and initiatives target working conditions which are known to be associated with retention; however, these initiatives have been poorly implemented or enforced, with few formal evaluations of such interventions.^5^ Moreover, current research is limited to the perspectives of specific professional groups and most are survey-based studies.^18^

In order to better address current working conditions, with a view to improving retention, this research was aimed at determining practical barriers and opportunities for change in the ED working environment as perceived by professional staff working in this environment. This will tooffer insight into the shared experiences, constraints and priorities of those working within the ED.

Enhanced understanding of these issues can provide a firm basis from which to shape, inform and underpin future policies and workplace initiatives, ensuring that practical barriers and opportunities for change are embedded in a way that optimises relevance and feasibility of implementation in the ED working environment.

### Study aims and objectives

This study sought to engage three core professional groups (doctors, nurses, advanced care practitioners; ACPs) who work within an EM context to better understand (a) primary concerns relating to working conditions; (b) perceived barriers to implementing change and (c) perceived opportunities and targets for change. Findings will be used to underpin key recommendations that are tailored to the needs of an over-burdened and under-resourced ED.

This qualitative study forms part of a larger collaborative project between the University of Bath and the Royal College of Emergency Medicine (RCEM), funded by a UKRI Policy Fund. The full recommendations relating to the four core themes are available on the RCEM website (Psychologically Informed Practice and Policy (PIPP) | RCEM).

## Methodology

### Design

This study uses a qualitative approach involving online focus groups in order to gain a rich and detailed understanding of participant perspectives and views, unrestricted by closed question responses. Focus groups offer the opportunity to gain an understanding of shared experiences and narratives, using a dynamic approach to the subject matter, allowing further probing for clarification and participant interaction for deeper insights. The COVID Clinicians Cohort (CoCCo) study^19^ was used to organise data into key categories; this model mirrors Maslow’s Hierarchy of Needs^20^ from a workplace perspective.

### Participants

To be eligible for participation, ED staff must have been currently employed in a UK NHS ED as either a doctor, nurse or ACP.

ACPs are a recently developed workforce of accredited clinicians who have received advanced training to expand the scope of their usual role (eg, paramedic, nurse), permitting them to take on additional clinical responsibility in the ED.

These three groups are core affiliates of the RCEM and represent the majority of the workforce in the ED. The ED setting was used as the focus (rather than all acute care settings) as this represents the core and central setting for EM.

### Recruitment and procedure

Online adverts and qualtrics survey links were distributed through social media (ie, Twitter) and RCEM communication channels using snowball recruitment methods. Profession-specific focus group interviews were conducted online using MS teams by two study researchers (JD, ER) using a semi-structured topic guide (see online supplemental materials). The guide was shaped by the scope of study aims and the current evidence base and explored difficulties in the work environment, impact of these difficulties, barriers and priorities for change. Focus groups were 60–90 min in duration and were recorded using encrypted audio recorders, transcribed and stored securely. Participants were given debrief information sheets following the focus group. Transcripts were not returned to participants and no repeat focus groups were carried out.

10.1136/emermed-2023-213189.supp1 Supplementary data

10.1136/emermed-2023-213189.supp3 Supplementary data

10.1136/emermed-2023-213189.supp2 Supplementary data

### Analysis

Directive content analysis was applied to the data.^21^ This analysis strategy was used to identify common themes from participant responses, using deductive codes by identifying key concepts from existing theory^19^ and prior research. Two researchers (ER, JD) read through each transcript, highlighting passages that could be categorised in the pre-determined codes. Any passages that could not be categorised within the initial coding theme were given new codes. Further coding was then conducted and this iteration was reviewed and updated. After coding was completed, initial notes from the focus groups were revisited to ensure all reflective notes were incorporated where relevant. Final themes were refined through an iterative process between JD, ER and EJ (qualitative analysis expert), with all stages of analysis reaching consensus agreement with regard to the content and labelling of codes and themes.

### Patient and public involvement

As this study focused on staff experiences in an EM workplace, a Clinical Advisory Group (CAG) was used in place of patient or public involvement. The CAG comprised of five clinicians working in the ED who advised on the scope and priorities of the study. This included two medical consultants, one charge nurse, one trainee and one specialty grade doctor. Of those, three were males and two were females. All CAG members were offered renumeration for their time.

## Results

Of the 117 total responses to the study advert, 16 respondents were eligible but not available to attend focus groups and 55 either did not consent or were not eligible based on their role and/or department. From the remaining 46 respondents, 13 of these could not attend or cancelled, leaving a final sample of N=33 (28% of total responses). Due to higher response rates from doctors, these focus groups were further grouped by grade; nurses and ACPs were grouped by profession only and were organised base on availability. There were 11 groups in total (see table 1). Participants were mostly female, and from a white British background. Ages were spread fairly evenly across the categories, except ages 35–44 which included substantially fewer participants.

**Table 1:** Participant and focus group characteristics

Characteristic n=33		n per group (% of total)
Professional groups	Medical staff groups	20 (60%)
	Consultant grade doctors	3
	Consultant grade doctors	3
	Postgraduate doctors in training	2
	Postgraduate doctors in training	4
	Specialty and associate specialist doctor	4
	Specialty and associate specialist doctor	2
	Consultant grade doctors—clinical lead role	2
	Nursing staff group	8 (24%)
	Nursing staff	6
	Nursing staff	2
	Advanced care practitioner group	5 (16%)
	Advanced care practitioners	3
	Advanced care practitioners	2
Gender	Female=24 (73%) Male=9 (27%)	
Age	25–34 years old=8 (24%) 35–44 years old=4 (12%) 45–54 years old=8 (24%) 55 years or above=7 (21%)	
Geographical spread	North East Scotland=1 (3%) North East England=2 (6%) North West England=1 (3%) East of England=2 (6%) West Midlands=3 (9%) South East England=3 (9%) South West England=11 (33%) East Midlands=2 (6%) London=6 (18%) Ireland=1 (3%) Missing=1 (1%)	
Ethnic origin	English/Welsh/Scottish/Northern Irish/British=28 (85%) Indian=3 (9%) White and black African=1 (3%) Any other white background=1 (3%)	

### Analysis

Following analysis of the qualitative data, four key themes were generated. These were termed: ‘culture of blame and negativity’, ‘untenable working environments’, ‘compromised leadership’ and ‘striving for support’. Data within these themes that were identified as ‘barriers’ or ‘opportunities’ for change were extracted (table 2). Illustrative participant quotes are identified by researcher codes, which reflect the profession and a recoded group number, to preserve anonymity.

**Table 2:** Primary concerns, barriers and opportunities for change

Primary workplace concerns	Barriers to changing working conditions	Opportunities for change
Culture of blame and negativity	Negativity and toxicity among colleagues ‘Outdated’ perceptions of clinical demand Expectations and frustrations from those we care for Lack of investment in staff development	Interprofessional valuing and respect Culture of care and shared responsibility Team cohesion Clearer lines of accountability Nurturing growth
Untenable working environments	Understaffing and high workload Unmet physical needs High-intensity workload Lack of autonomy over working patterns The shifting nature of work	Viable staff ratios Access to hot food and rest spaces Protected study time Self-rostering A department that is well-resourced and fit for purpose
Compromised leadership	Team expectations of their leaders Realities of working as a clinical lead Bridging the gap between the ED and executive management	Compassionate leadership Role clarity Shared resources Access to mentors and coaches Protected time to do the job Access to leadership training and support
Striving for support	Access to support Mental health stigma	Protected time to access support Prioritisation of well-being in the ED Embedded psychology Peer-to-peer support Levels of care, tailored to need

### Culture of blame and negativity

When asked about the most difficult aspects of their working conditions, participants commonly reported a culture of blame and negativity in the ED. The work culture not only felt unsupportive and ‘toxic’ but had a marked effect on well-being. Participants described a culture which was quick to blame rather than support:

You worry about making a mistake, and if you did make a mistake who would have your back. (ACP, G7)

You very rarely get anyone saying that was a good job. (SAS doctor, G8)

This was particularly felt top-down, where those in management position were perceived to take an unsympathetic view of extended waiting times and unmet targets, despite the tangible constraints of operating at overcapacity and ‘exit block’, problems that participants perceived to be out of their control. Participants in all groups indicated that the negative culture instils anxiety over how they might be perceived by peers, but particularly by senior colleagues:

That’s a classic example… she’s a senior member of the team, really knows her job…. She was quite critical really, in a very negative way about how you managed that patient. (Nurse, G11)

Some participants reported senior colleagues having unrealistic expectations of the more junior staff, with little consideration of the increased pressures that have arisen in recent years:

It’s ridiculous to compare the needs, even for our senior colleagues who were registrars five years ago, the reality of running the department overnight is not the same as it was then. (SAS doctor, G1)

Existing structures and working practices of the NHS were described as ‘archaic’ and ‘old fashioned’, leading staff to feel blamed if they could not cope with the pressures and disempowered to seek support due to the expectation that they should be ‘unbreakable’ (Trainee, G9). Participants also voiced that they were unclear on lines of accountability, who to approach for what problem. This barrier to escalating their concerns was further compounded by the belief that both clinical leadership and higher management were generally overburdened and unreceptive to discussions on workplace concerns.

Increasing pressure and longer waiting times were described as driving antisocial behaviour from patients, exposing staff to risks to physical and psychological well-being:

So the long wait causes verbal or physical violence and aggression, which has a massive impact on staff well-being. (Nurse, G11)

Participants highlighted the desire to be supported to learn from difficult experiences and develop in light of them, suggesting that a simple checking in on how individual staff members are progressing would be well received and beneficial to well-being:

We have intermittent debriefs… but it’s not every time. It doesn’t necessarily need to be every time, but it’s not as frequent as it should be. Even if it is just ask are you okay? (Trainee, G5)

Interprofessional respect and development of a more empathic culture of shared responsibility were flagged as key opportunities for change that would support better team cohesion:

We need to change how we speak and respect each group, and we need to try and understand each other’s point of view, and if we could get better ways of working, but just talking to each other about what are my problems, what are your problems, why is this stressing you, what’s stressing us, how can we work together to do that. (ACP, G2)

Findings suggest that EM professionals are confronted with outdated perceptions of clinical demand from within teams and systems, with unrealistic expectations which compound a blame and shame culture when expectations are not met. Operating within this chronically under-resourced system was framed as compromising workforce well-being and risking burnout, yet participants indicated that simple interventions such as check-ins, clearer lines of accountability and a more civil and respectful culture would offer key opportunities for growth and sustainability even in the face of a staffing crisis.

### Untenable work environments

The complex work environment within the ED was described as being of significant concern, compromising care and leaving staff feeling undervalued due to basic needs being unmet. Participants frequently reported poor quality or inadequate facilities, such as provision of toilets, lockers and changing rooms, hot food only available within limited hours, poorly functioning IT systems and rest spaces being in a different building.

So you’re just basically sharing (toilets) with the patients. In the urgent care centre there’s two toilets for the whole of the department in there, often one of those is broken…and not enough lockers for every member of staff. (ACP, G2)

Stuff like working computers, a consistently working POD system… those little things I think make a bigger impact on your life than how many people come in through the front door. (Trainee, G5)

A lack of physical space for administrative tasks was highlighted by many clinical staff, being described as ‘woefully inadequate’ (ACP, G2). Wards were described as ‘unfit for purpose*’* (Nurse, G11), which was attributed, in part, to higher management lacking understanding of the needs and practices of the ED. One example highlighted the long-term impact of ED workspace changes that were not fit for purpose:

…it was clear that no clinical staff had been involved. Doors were in the wrong space, no sinks in the right place, not enough storage, poor flow, poor layout (ACP, G2)

Existing rest spaces or staff rooms were reported to be taken over to provide more clinical room, limiting the space for staff to change, rest and decompress.

The nurses were getting changed in a corridor, now they seem to have a cubicle they can get changed in. But the facilities for the same trust are really very different. (Nurse, G10)

This was perceived to be particularly important due to working in the high-pressure environments of a crowded ED, where staff voiced concerns regarding the sustainability of working with a high workload safely without private spaces.

EDs were perceived to be more busy, for reasons associated with shifts in societal expectations and perceptions of the scope and role of ED:

Go back ten years ago in the emergency department and people would try their best at home, would take painkillers, will see how it goes, not wanting to trouble A&E, but seems like now it seems like A&E is the open door for everybody just to come in with everything. (ACP, G7)

Participants used emotionally laden language when describing the intensity of the workload itself, with parallels drawn between being at war and working on the NHS frontline, where staff worked under similar levels of intensity but longer term and without rest.

…when people are deployed (in the forces) they are deployed for 6 months…because that 6 months is intense, it’s intense on your body, it’s intense on your mind, it’s intense on your family, it’s intense on everything about you, and that’s while you were deployed for 6 months, and then there’s some recovery time coming back. (Consultant, G4)

Comparisons were also made to the sinking of ‘the Titanic’:

There is the jollying everybody along, being the redcoat on the shift, cheering everybody up, saying everything is going to be okay, but feeling like you’re just rearranging the deckchairs on the Titanic (Nurse, G10)

The impact of a consistently high workload was described as being compacted by a lack of agency and autonomy over working patterns, which was perceived to be related to non-clinical staff making decisions about shifts without understanding the inherent pressures:

The people who control our rotas are… her job is a rota co-ordinator, she works in an office, she is administrative, and the person who signs that off is the manager for the department, again non-clinical, and getting leave is a nightmare, it’s awful. (Trainee doctor, G5)

Consultants identified that there were limited options to reduce workload when approaching retirement, and they did not necessarily feel well-equipped to continue operating under high pressure and for long hours. Those in training posts reported insufficient time to meet requirements or study due to workload, influencing both career progression and confidence in the role.

You are getting no progression because you’re not getting your training, and I know that personally in the last year I made my decision that I will not continue to work clinically, I will step back in the next few years because there’s… why would I stay doing something that there’s no reward for? (Nurse, G11)

Participants agreed that there was both a need and an opportunity for the ED to be a ‘nicer place to work’ (ACP, G2). Specific suggestions included a full staffing quota, ensuring staff are adequately rested to return to work and the opportunity for peer support:

My top three things would be coming on with a full staffing quote so you know there’s no gaps in the rota, so you’re all there. Everyone is well rested and ready for the shift, just being able to talk to each other on the shop floor and being quite open with each other on how everyone is feeling. (ACP, G7)

Many of the suggested changes directed at making working conditions in the ED more sustainable related to basic needs such as being able to take breaks, access healthy food and functioning IT when needed:

…having those opportunities to go off and have a five minutes when you need to, to be able to continue your shift. (ACP, G7)

It would be really nice to be able to have some healthy nice food in the department. (Nurse, G11)

As more and more of our job goes electronic, electronic notes, electronic prescribing, actually having IT systems that are fit for purpose, everyone has access to (Trainee doctor, G9)

Self-rostering was frequently mentioned as a positive experience for participants and a useful avenue to help participants to deliver better care and improve well-being:

One day off between a set of shifts is not enough to decompress and be re-energised to start back on your next set of shifts. So I think the rota, we have moved to a more self-rostering method now, and I think that’s helping with staff well-being, especially in our team. (A7)

Overall, working in existing ED environments was described as ‘untenable’ and ‘unsustainable’ in terms of both the working environment and the lack of agency and autonomy over high-intensity workloads. Many of the problems and solutions relate to provision of resources to meet basic needs, many of which are subject to professional and NHS regulations; however, due to pressures this is not being implemented.

### Compromised leadership

Clinical leads in the ED were perceived to hold responsibility for setting the tone for culture and behaviour in the ED, leading by example:

And you lead by example as well, so if your consultant in charge is not taking a break you feel like you can’t ask to take a break. It’s the same with the nurses, if the nurse in charge is not taking a break then a lot of the junior nurses won’t come and ask for a break because again you’re guided by the leadership aren’t you? (A7)

The clinical lead in the ED is a key conduit for change, from a cultural and environmental perspective especially. However, participants expressed frustration about feeling that their voices were not heard or valued outside of the department, in part due to clinical leads being reluctant to escalate their concerns due to the discrepancies between clinical priorities within the ED and the priorities expressed by trust level executive management:

You’ve got the clinical side, and we are to one degree or another worried about the patients, and then you have got the management side and they are worried about figures, times or money, and those two things don’t really mesh together (ACP, G2)

Yet, within the EDs, leadership was described as being poorly supported in terms of protected time to train and deliver the role fully. Consultants voiced reluctance to take on a leadership role due to lack of ‘visible leaders’ to provide inspiration or exemplar: ‘There is no one for us to look up to, to lead us’ (Consultant, G4), ‘We need compassionate leadership’ (SAS doctor, G1).

A lack of definition or clear understanding of what the clinical role entailed was reported to make it difficult for clinical leads to be effective in their role:

People tell you that you’re there to lead, and you’re like I know but what does that mean? And then you don’t know if you’ve got to go to all these meetings, which ones you really need to go to, which ones can I not go to, also for me I do the job on my own. (Clinical lead, G6)

Participants emphasised they need a ‘clear definition of what the college would see the role to be, and how much time they would expect it to take of your job*’* (Clinical lead, G6). Any possibility for growth was hampered by a lack of training or support from colleagues to help with even the practicalities of the role (such as recruitment and personnel management):

I have literally started last week on a leadership course that’s been for other clinical leads in the organisation. But I feel a bit could have done with this maybe earlier. But that’s more about your leadership qualities and conflict resolution, it’s all that side of it as opposed to the actual practicalities of the job. (Clinical Lead, G6)

When considering possible solutions to these difficulties, participants suggested that an accessible time to do the job and an online repository may offer an opportunity to share resources, learn from one another and foster development:

I think sharing all the stuff we shared on the WhatsApp, trying to share stuff, so how to write a business case, what you need to do. (Clinical lead, G6)

I should be doing work at a time I am getting paid, so you need to give me that time. (Trainee doctor, G9)

Mentorship was also deemed to be important for successful delivery of the role:

I think personally as leads and stuff we should all have some kind of mentoring type…Supervision, that’s the thing, we don’t get any. (Nurses, G10)

Participants described having difficulties feeding into emerging issues to address unmet need, blocked from communication with leaders by ‘layers of bureaucratic sediment’. This was compounded by the career trajectory of NHS management, where often those in post would swiftly move on for promotion.

Overall, clinical leadership within the ED was described as compromised, unsupported and, ultimately, a key barrier or missed opportunity for change in culture and working practices in the ED. However, there were clear indications of opportunities for growth and change, including a need for compassionate leadership, shared resources, time to do the job and mentorship.

### Striving for support

This final theme encompasses the concerns raised by participants regarding well-being and staff support, specifically the barriers to accessing well-being support and their preferences in relation to what changes are likely to improve their well-being. Common barriers included having to attend support or well-being services during time off, with the scheduling of support geared to a ‘nine to five’ non-clinical workforce (ACP, G2). Mental health stigma in the ED was also cited as a key barrier.

I think for me it still feels like a bit of a stigma about saying I am struggling what should I do next. (Nurses, G11)

There’s nowhere that I can express how I am feeling or even understand how I am feeling. (Consultant, G4)

This was reinforced by well-being not being viewed as a priority, with team check-ins or formal appraisals described as having ‘nothing in there about wellbeing’ (Clinical lead, G6), despite suggestions that simple well-being check-ins would suffice.

Participants suggested that support should not be purely accessed after the fact but something that should be prioritised and routinely available to staff to safeguard mental health:

… psychological support…it shouldn’t be something that we access when there is a problem, it should be something where we go well every month on a Friday at this time I go and talk to someone about what I have seen. (Trainee, G9)

Participants’ lack of understanding about which services were being offered was raised by many, with participants often able to list services available, or where the staff support centre was based, but not how or when one might access them. This offers a key opportunity for collaboration between staff support services and the ED to develop clearer pathways or a clear role for a departmental well-being lead.

Peer support was consistently highlighted as a highly valued resource that should be considered part of supportive culture ‘gives you somebody else to share the load with, and not be that single voice’ (Trainee doctor, G9). However, limited physical space and time to engage in peer activities were cited as barriers:

Well yeah it would be lovely to sit down and chat with my peers, apart from the fact that 1) we’re constantly busy, 2) we don’t have anywhere where we can sit and have a confidential gas. (SAS doctor, G8)

Overall, accounts suggested that existing support was largely unfit for purpose, and where it was easy to access (such as peer support) and available, it was often incompatible with ED working practices and within a culture where seeking support was often stigmatised.

Some participants expressed that having a psychologist embedded within the department was highly valued as a resource, particularly the different levels of support dependent on need:

…(during the pandemic) we setup weekly drop-in sessions with the psychologist… and it was really great for a lot of people to be able to drop-in, and then that led on to having one to one for people who felt they needed that, and also within ED we had a psychologist come round to our supervision when we needed them. (ACP, G7)

Participants reflected that psychological input introduced in response to the impact of the COVID-19 pandemic was highly valued. While many were open to discussion about their mental health and well-being, for many, stigma still permeates the ED culture and is further compounded by poor understanding and communication of available resources. Appointment of well-being leads, more value placed on well-being (including informal peer support) and routine access to psychology are suggested as opportunities to make strides towards improved well-being.

## Discussion

This study identified four key themes describing the difficulties in the ED work place. Working culture, physical working environment, pathways to care and leadership represent the core workplace concerns within our sample. These issues were perceived to play an instrumental role in their ability to sustain good working practices, well-being and, importantly, their intention to leave. Participants identified key barriers and opportunities within their work contexts which resonate with existing research and policy and can be used to shape the future policy and research development.^22^ ^2 5^ These findings act as a basis for the development of specialty-specific targets for change that are aligned with the views and voices of those working in this working environment and also take account the barriers and opportunities faced in the fast-paced unique environment of the ED. For a full set of EM-specific recommendations to underpin change across all of these four areas, see the Psychologically Informed Practice and Policy (PIPP) recommendations (https://rcem.ac.uk/workforce/psychologically-informed-practice-and-policy-pipp/)

Several of our findings have been noted in previous studies, particularly the role of culture, environment and access to support.^22^ Most of the research examining factors associated with working conditions and retention in EM are profession specific^3 6 18 19^ and are not readily generalisable to other professional groups in the ED. However, our study included doctors, nurses and ACPs from which emerged common cross-cutting themes affecting all of these professions working in the ED, themes which are consistent with the broader literature^9 10^ but specific to the EM working environment.

As reflected in the work by Darbyshire *et al*,^5^ the nature of the problems described were systemic; the workplace challenges were interrelated and appeared reciprocal in influence, arguably maintaining one another. The cyclical nature itself proves a key barrier to change, which raises the question: which is the primary target to effect most change? Leadership has a pivotal influence across these themes and is unequivocally vital to workforce transformation; however, this is an area that has been largely neglected in EM, with very little research seeking to develop or evaluate leadership interventions in this environment. Indeed, there is an assumption that leadership naturally develops over time and is fully formed on appointment to the role.^23^ However, leadership within the ED is particularly complex and demanding due to the range of competencies required (clinical, managerial and administrative)^23^ and the high-pressured environment within which this role needs to be delivered. This warrants tailored training and support to fully succeed. In settings where the nature of the work is unpredictable and at times clinically critical, leadership is pivotal to patient outcomes and team functioning,^23 24^ which are particularly crucial in the ED setting. Leadership has the potential to be a powerful driver in workforce transformation, cultural change^25^ and team functioning within these highly skilled, professionally interdependent teams.^26^ To fully harness the capacity of leaders as agents of change, those in leadership positions must be sufficiently skilled,^27^ feel supported to act on important issues^27^ and have time to do the job. Yet, participants in this study reported poor role definition, lack of training and absence of protected time to deliver the role. This was compounded by blurred lines of accountability that led to impotence to effect change.

### Implications

The development of leadership in EM should now be a primary focus. There are clear steps that can be taken to begin to mobilise and maximise the pivotal influence of leadership in effecting change, across government, professional, organisational and individual levels.

On a public policy level, there has been a rapid growth of government level publications and resources to recognise the role of leadership as a conduit to better patient and team health.^28^ However, recommended leadership training is often generic and never mandated. This is surprising given the clear links with patient safety and team functioning.^23 24^ Leadership training in healthcare should be mandated by government bodies, not least due to links with patient safety.^29^

Significant work has been undertaken by RCEM to integrate and embed mandatory leadership training into the training curriculum for EM trainees, without which they cannot progress. While this demonstrates forward thinking and some future-proofing for the medical profession, it cannot cease at this point, it must be supported with continuing professional development post-training. The relevant professional bodies provide access to good quality leadership training such as the RCEM EM Leaders Programme and the RCN Leadership Programme, however, this is largely online without protected time to access or support development. More work is needed to ensure leadership training is visible, supported as part of a workplan, and a priority area championed by all relevant professional bodies.

Further work is needed to ensure that leadership competencies are introduced at an early stage of training^23^ so the necessary skills are embedded and cultivated on the pathway towards and within leadership roles, rather than ad hoc when necessity dictates. This falls to both training and professional bodies to work together to ensure that theory-driven leadership is a core part of the teaching curriculum, with mentorship and practical resources (such as role definition, a personal development plan, human resource support) to complement and facilitate the necessary continuing professional development throughout a clinical career. Responsibility then moves to the employing local NHS trusts to support the development of those individuals within leadership positions. It is at this level that ED clinical leads and their teams can harness their influence; local NHS trust policies are driven by guidance from government and professional bodies, however, they have the power to shape local policy and mandate change in view of the needs of a service. We summarise key recommendations to underpin change at a local NHS level in Box 1.

Box 1 **Key leadership recommendations for local NHS trust level commissioning**

- Those in leadership positions should be supported to attend leadership training as part of their workplan, within their workplace hours. This would include top-up training and training assignments.
- Support to engage with a leadership mentorship or coaching programme as part of their workplan, with a view to continuing professional leadership development and creating safe spaces to problem-solve, reflect and seek support.
- Access to the consultation service within the local NHS staff support services.
- Appointment of a designated ‘Wellbeing Lead’ with protected time and support to deliver the role.
- Clear description of roles and responsibilities, to include protected time dedicated to undertaking additional responsibilities associated with a leadership role and a professional development plan that is reviewed annually.
- Support to engage with the EM clinical lead network in order to access resources to support the delivery of the role and access peer support when necessary.
- Clear lines of accountability at an NHS organisational level with identified pathways to escalate concerns.

EM, emergency medicine.

Appointment of well-being leads within the ED, as outlined in the RCEM PIPP recommendations^30^ and other key documents,^22^ is also a key step towards workplace transformation through leadership; however, it is imperative this role is also supported with protected time and development. A well-being lead with a clearly defined remit and role would play a pivotal gatekeeper role in encouraging attitudes towards well-being in the ED by delivering ‘warm handovers’ and well-being initiatives, such as informal check-ins, staff team activities (ie, safety huddles), and well-being surveys.

On an individual level, those in leadership positions are more likely to succeed by harnessing the influence and opportunity that accompanies the role, identifying and taking inventory of challenges and barriers, clarifying lines of accountability to drive forward change and advocating for the needs of their team. Two mechanisms by which leadership bears the greatest influence include leading and prioritising a continuous cycle of quality improvement (eg, autonomy over work patterns, access to rest spaces, patient flow, taking steps to address the diversity gap) and role modelling of positive professional behaviours.^26^ The latter includes compassionate and inclusive attributes but also speaks to the necessity to meet basic needs: taking breaks, adhering to annual leave, destigmatising views on mental health and openness to learning and change. Those in leadership roles should be encouraged to engage with the leadership networks, broadened to encompass a platform or virtual environment (ie, repository) to share and access resources and be granted access to leadership consultation with the well-being team as and when necessary. Those in leadership positions should also be provided with clear referral processes and internal professional standards to help address any incivility, including bullying, harassment and issues of inclusion. This would help promote a culture of care and interprofessional valuing and respect, improving team cohesion.

Finally, it is imperative that lines of accountability are clear for those in a leadership position. While many NHS trusts differ in their management structures, each trust will have communication pathways to divisional and executive management leadership teams. In order to drive the full potential of leaders to action change through these mechanisms, it is fundamental that pathways from ‘shop floor’ to the chief executive are clear and opinions and concerns of ED leadership are welcomed.

Flow through the ED, staff ratios, pay and pension structures are of course prime targets for change and where the current high-profile focus lies. However, leadership is a key conduit to change and those with mandatory powers must now move to recognise this in order to unlock the full potential of this role.

### Limitations and future directions

There are inherent limitations in the small size of some of the participant groups, and as such the views and opinions expressed cannot be considered transferable across their respective professions. While many prospective participants did not proceed to focus group meetings due to last minute requests to cover shifts, the participant pool was comfortably within the bounds of what is acceptable for a qualitative study.

Findings should be interpreted in light of the sample consisting mainly of white women, therefore the views of males and minority groups may not be fully represented. Doctors made up a higher proportion of the final sample; this may be a consequence of using RCEM communication channels as a primary recruitment method, which has more members registered as doctors than nurses. As not all professions working in ED were included (eg, physiotherapy, psychology) it is possible that additional themes or differences might have been missed.

The geographical spread reflects a broad reach; however, there was a preponderance towards the South West, where the research was conducted. While none of the interviewees were known to the research team, those in the South West may have been more exposed to recruitment drives through mutual connections.

The development and testing of leadership training and packages should be a priority for professional bodies and at organisational level. This should take account of the overlapping and competing competencies required of ED leadership, including managerial, administrative and clinical components and the high-pressured context within which these skills are required.

## Conclusion

This study identified key themes in understanding workplace concerns in the ED, and their associated barriers and opportunities for change. Leadership in EM should now be a primary focus, with further investment and support to target the development of leadership skills early on in training and provide protected time to refine these leadership skills and qualities across the working lifetime. This will serve to harness the pivotal influence of leadership in EM, which, if properly supported, holds the potential to act as a conduit for change across all areas of focus.

## Data Availability

Data are available upon reasonable request. Requests go to the corresponding author - Jo Daniels (j.daniels@bath.ac.uk, University of Bath, UK). De-identified participant data can be made available upon reasonable request.
