# Supplementary material for: Perceived barriers and opportunities to improve working conditions and staff retention in emergency departments: a qualitative study
Source: Emerg Med J. 2024 Jan 9;41(4):257–65. doi: 10.1136/emermed-2023-213189 (PMC10982618; doi:10.1136/emermed-2023-213189)
Supplement: Supplementary data [file emermed-2023-213189supp001.pdf]

Focus group topic guide

1. What do you find most difficult about your working conditions?
   a. Are there any specific challenges that are relevant to your profession that are not felt by others?
2. How does this affect you? (home life, work aspirations, mental health)
3. Is there anything you feel is missing in terms of support for these issues?
4. How could this best be addressed?
   a. What are the top priorities when beginning to address these issues?
5. What barriers are there, in your workplace, to implementing this change?
6. Thinking about the triangle (show model) what areas do you feel are most neglected where you work?
7. How will you know if things have improved enough?
   a. What tangible change do you hope to see?
8. How will this improvement show in you?
9. Tell us what is going well?
