# Supplementary material for: Perceived barriers and opportunities to improve working conditions and staff retention in emergency departments: a qualitative study
Source: Emerg Med J. 2024 Jan 9;41(4):257–65. doi: 10.1136/emermed-2023-213189 (PMC10982618; doi:10.1136/emermed-2023-213189)
Supplement: Supplementary data [file emermed-2023-213189supp003.pdf]

University of Bath · NHS North Bristol NHS Trust · Royal College of Emergency Medicine · UK Research and Innovation

"We are just rearranging deckchairs on the titanic"

Retention of staff in emergency medicine is at crisis level

The PIPP project looks at the issue of retention in emergency medicine, taking a psychologically informed approach to understanding and identify key targets for change.

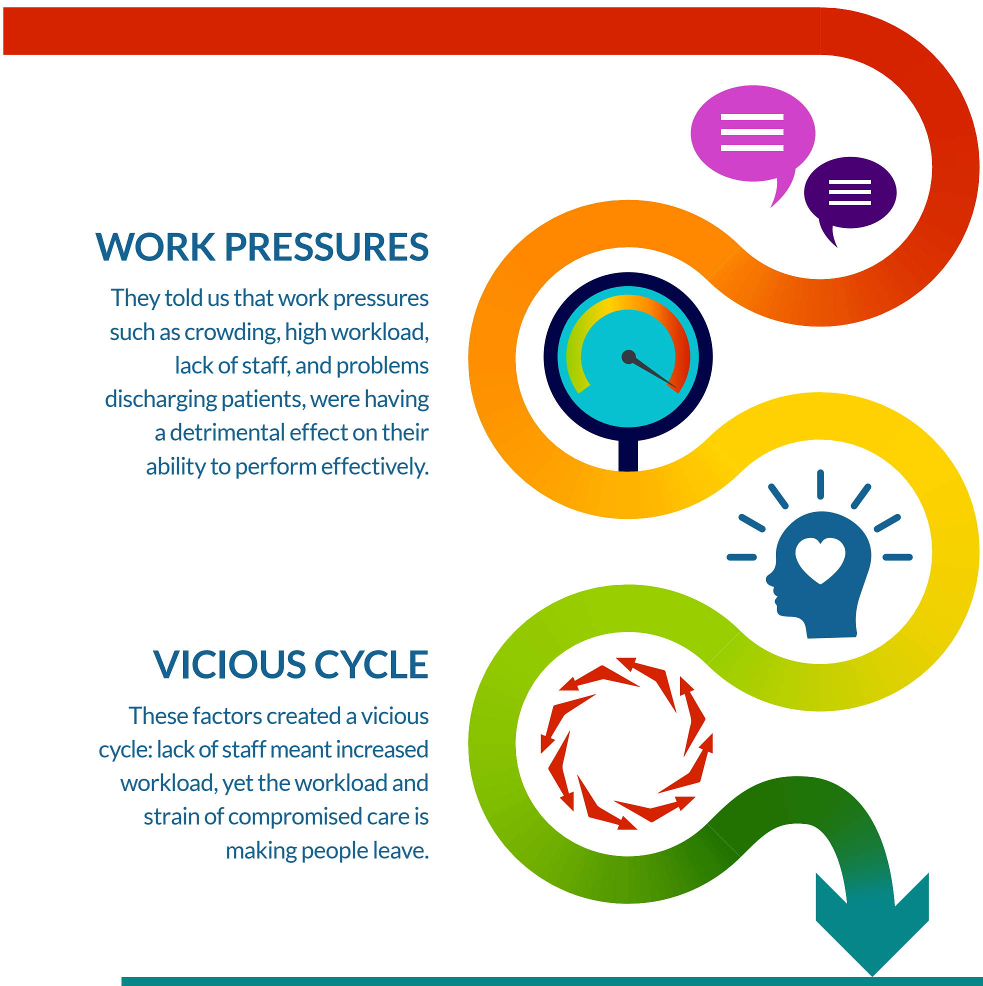

FOCUS GROUPS

We used focus groups to speak to clinical staff working in EDs, asking them how they felt about their work.

WORK PRESSURES

They told us that work pressures such as crowding, high workload, lack of staff, and problems discharging patients, were having a detrimental effect on their ability to perform effectively.

PERSONAL BURDEN

These work pressures affected how they felt about themselves, their jobs, and their ability to deliver good care. This made them feel unhappy at work and consider leaving.

VICIOUS CYCLE

These factors created a vicious cycle: lack of staff meant increased workload, yet the workload and strain of compromised care is making people leave.

WHAT CHANGES ARE NEEDED?

We asked them what changes would make the biggest difference to their working lives. **They said:**

AN ENVIRONMENT TO THRIVE IN

- Viable staff ratios
- Access to hot food
- Adequate rest places
- Protected study time
- Self-rostering
- A department that is well-resourced and fit for purpose

CULTIVATING A BETTER CULTURE

- Culture of care and shared responsibility
- Inter-professional valuing and respect
- Team cohesion
- Clearer lines of accountability
- Nurturing growth

A TAILORED PATHWAY OF CARE

- Prioritisation of wellbeing in the ED
- Embedded psychology
- Peer-to-peer support
- Levels of care, tailored to need
- Protected time to access support

ENHANCED LEADERSHIP

- Clear compassionate leadership that is visible and supported
- Access to leadership training and support
- Role clarity and shared resources
- Access to mentors and coaches
- Time to do the job

We know what is needed to make the ED a better place to work, but we need to act now – before there is no one left to take care of those who most need it most.

#PIPP
