# Supplementary material for: Perceived barriers and opportunities to improve working conditions and staff retention in emergency departments: a qualitative study
Source: Emerg Med J. 2024 Jan 9;41(4):257–65. doi: 10.1136/emermed-2023-213189 (PMC10982618; doi:10.1136/emermed-2023-213189)
Supplement: Supplementary data [file emermed-2023-213189supp002.pdf]

January 2023

Psychologically Informed Policy and Practice Development (PIPP) Project

Key Recommendations for Retention and Workforce Wellbeing in Emergency Care

This document details specific evidence-based recommendations relating to four key areas identified as prioritised targets in emergency care workforce development: 'an environment to thrive in', 'cultivating a better culture', 'a tailored pathway of care' and 'enhanced leadership'. The recommendations are detailed, supported by evidence, existing guidelines and new empirical data, and are specific to the needs of the emergency care specialty. The PIPP projects investigated current workplace concerns, barrier to change and opportunities for development and growth. These recommendations are an extract from a full project report, which is available from j.daniels@bath.ac.uk. The executive summary is available on the Royal College of Emergency Website (RCEM) here and the brief PiPP study animation overview can be accessed here.

Recommendation 1: Creating an environment to thrive in

ED clinical staff in the PIPP study outlined a drive to develop and learn in their working environments. They should be provided with all that is necessary to thrive in the workplace, which will influence performance, patient care and job satisfaction. This forms part of the 'A – Autonomy and control' aspect of the General Medical Council's (GMC) 'ABC' core needs for NHS staff wellbeing and motivation at work. In line with our recommendations, 'ABC' (GMC, 2019) suggest all healthcare employers should provide adequate rest spaces, protected time and the tools to do their job effectively. They also recommend that clinical staff are given the opportunity to feedback on how their work is organised and delivered with a focus on learning (GMC, 2019). These recommendations mirror recommendations made in the Royal College of Nursing's (RCN) Healthy workplace toolkit (RCN, 2021[2]). The following further specifies the core areas to address in order for PIPP participants to thrive at work.

a. Viable staff ratios

Minimum staff ratios are vital to the wellbeing of both staff and patients. Statistics from RCEM suggest that there is a current shortfall of 2,000-2,500 whole time equivalent (.wte) consultants in the UK (RCEM, 2021); a key factor identified by participants to influence wellbeing and retention. RCEM have worked with the RCN to publish nursing workforce standards (RCEM & Royal College of Nursing, 2020), which outline minimum staff numbers required for a type 1 emergency department. These standards must be adhered to, in order to achieve safe staffing levels and decrease the burden on existing staff; it is vital that emphasis is placed on the patient safety implications.

b. Access to hot food

Access to healthy hot food should be available to all staff at all times during shift working, including out of hours during a night shift, whether this is access to a canteen or through provision of means to purchase and heat food. Access to basic food provision as set out by the NHS Staff and Learners' Mental Wellbeing Commission Report (Health Education England, 2019) is a minimum requirement in all EDs, and has been a key issue identified across our body of research (Daniels et al. 2021; Harris et al., 2021) and elsewhere (Boyle, 2022; Department of Health and Social Care, 2020). The Mental Wellbeing Commission Report highlights that wellbeing at work needs to be addressed strategically but it is often

the simple things, such as access to hot food, that makes the biggest difference. This was echoed by the clinical staff who participated in the PIPP and also the CoCCo project.

The provision of hot and freshly prepared food 365 days a year, including extended meal hours, is included in the British Medical Association's (BMA) Fatigue and Facilities Charter (2018). The charter also suggests that in the absence of catering facilities, hot food should still be readily available through microwaveable meals or similar arrangement. Other models, such as the Psychological Needs Assessment Tool (2021), also outline the importance of access to hot food, alongside other physical needs, as a key element for psychological safety for health and social care staff. It is common sense and supported by the evidence that without adequate sustenance, work satisfaction and performance will be affected; this in turn influences patient safety.

c. Protected study time

All clinical staff who have educational requirements should be supported to complete their coursework, which is monitored by the relevant educational provider and line manager. It is unacceptable to ask/require staff to complete mandatory study in time off duty, when rest and recouperation is essential. This applies to all professional groups, such as ACPs and SAS doctors, trainees and nurses. Data from the PIPP project suggests that clinical staff, particularly those in training and those in enhanced roles, are inundated with clinical work to maintain safe service provision. This erodes the 'protected' time for training and educational requirements as they are feeling placed in a difficult situation to either compromise care and 'let the team down', or continue with their studies. However, continuing education and professional development are also vital to patient safety; all planned teaching and training should go ahead as planned. The Care Quality Commission (CQC), as part of their departmental appraisal, requires healthcare services to provide protected time to staff to complete their training to a good standard and given opportunities to develop, so they are able to deliver safe and effective care (CQC, 2018).

Protected study time should form part of an educational contract and/or annual job planning and appraisal. Protected time and workload should also be considered when establishing portfolio expectations for trainees and CPD recommendations (RCEM, 2017[2]). Recommendations and lessons from best practice in the SAS development and retention programme should also be considered for SAS grade doctors to ensure they feel valued and supported in their professional development, particularly for the Certificate of Eligibility for Specialist Registration (CESR) training route (HEE, 2018).

These recommendations fall within the remit and responsibility of organisations such as RCEM, RCN and HEE who implement this guidance (HEE, 2020), however RCEM do not have mandatory power, unlike HEE. HEE has previously launched pilots in 2020 to improve protected time for professional development and offering additional learning pathways outside of their clinical specialty (HEE, 2020), however, this needs to be rolled out and implemented nationally, if shown to be effective.

d. *Adequate rest places*

Rest is vital for all staff, especially those on extended hours shifts. A rest space(s) must be provided which offers comfortable chairs and a space to lie down that is provided solely for the use of rest and decompression, not family spaces or patient provisions. This is in addition to a staff room which should be used for breaks, eating and socialising, and is in line with the HEE Mental wellbeing commission report (2019), which outlines the necessity of space to socialise, share experiences and rest. The NHS People Promise outlines safe rest spaces as essential to deliver the best care to patients. The requirement for adequate rest facilities also forms a key element of the NHS Health and Wellbeing Framework (NHS Employers, 2021), however, many clinical staff in this project (and also in Daniels et al. 2021; Harris et al., 2021) found that rest spaces were not in place or were not adequate.

Daniels J, *et al. Emerg Med J* 2024; 41:257–265. doi: 10.1136/emermed-2023-213189

e. Self-rostering

While already implemented in some sites, self-rostering should be introduced in all EDs to allow all staff and professional groups to engage with their lives fully and gain balance and autonomy in their lives. It must be ensured that departments are equipped with the specialised software for self-rostering to reduce barriers to access. This is in line with RCEM publication CARES policy and EMPOWER campaign. Autonomy is vital to the wellbeing of staff; a sense of control over work life is key to wellbeing and job satisfaction (GMC, 2017). Self-rostering also reduces the likelihood of overworking, contributing to a better working culture and improving work-life balance (Barrett & Holme, 2018). These issues are highly relevant to retention.

f. A department that is well-resourced and fit-for-purpose

All departments should take inventory of the resources required to support a fully functioning team. This includes provision of efficient IT facilities, personal protective equipment, spaces that are protected for writing clinical notes. Emergency departments are dynamic in nature and this poses unique environmental challenges; designing an efficient emergency department requires collaboration from multiple stakeholders, including leadership, clinical staff and executive management. All clinical staff should be encouraged to report and/or escalate concerns regarding physical resources and environment. More specific guidelines for what constitutes adequate resourcing has been produced by the Australasian College of Emergency Medicine (ACEM), which can be reviewed for use and implementation in the UK (ACEM, 2014). There is currently no UK guidance of this nature; this would fall within the remit of RCEM or RCN who are recommended to develop such guidance. In a recent survey of 1,663 responses, representing 192 healthcare organisations, no UK ED Electronic Health Record system met the internationally validated standard of acceptable usability for information technology (Bloom et al. 2021). Having systems that are inefficient and difficult to use can contribute to staff burnout, threats to patient safety and unfulfilling working life, by costing staff time and resources which could otherwise be put into patient care (Lee & Mylod, 2019). Access to efficient IT systems was a highly cited frustration in the focus groups; participants indicated an emotional impact (frustration) which interfered with their functioning, and a patient impact due to the extended time required to complete notes or input data, rather than treat patients.

Daniels J, *et al. Emerg Med J* 2024; 41:257–265. doi: 10.1136/emermed-2023-213189

Recommendation 2: Cultivating a better culture

Culture has a far-reaching influence and is known to affect job performance, wellbeing and job satisfaction. The culture in the ED was purported to have improved but was commonly labelled as 'toxic' (see figure 2 for word cloud derived from unsolicited descriptions of the ED culture). As reflected in business management 'culture eats strategy for breakfast'; the problematic culture, is likely to impeded positive change in other dimensions, and must be seen as a vital target and serious problem, rather than a 'softer' issue.

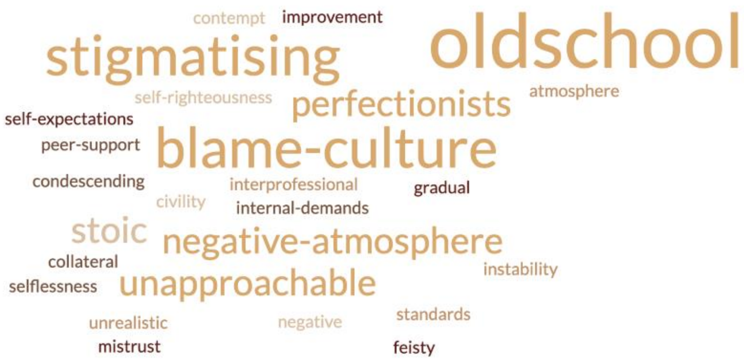

Figure 2. Culture word cloud

a. *Culture of care and shared responsibility*

While there are campaigns focussed on respect and civility which are reported to have gained positive impact (PIPP participants), an overarching approach to culture change in the ED is necessary to embody a true culture of care, i.e. shared responsibility for wellbeing as a team and for one another. As indicated in the NICE (2022) guidance, a proactive approach to wellbeing is vital, and culture is a key element within that. Steps need to be taken to cultivate a culture of care and shared responsibility, which include:

- Supporting adherence to rest breaks and annual leave entitlements – most powerfully done through shift planning and positive role-modelling of leaders
- Appointment of wellbeing champions, tasked with leading of wellbeing related issues (see recommendation 3)
- Increasing positive feedback/praise as part of a normal culture; finding innovative ways to give meaningful positive feedback outside of (but including) formal processes
- 'Warm handovers' where staff are supported to access the help they need - ensuring that recommendations to access support are followed up, retaining shared responsibility and accountability
- An RCEM campaign of care: co-creation of 'cultureED' campaign which amplifies the positive attributes required to work within this high-pressured stimulating field, focusses in on a culture of care, and seeks to name and eradicate undesirable behaviour.

The NHS Health and Wellbeing framework identify 'relationships' as a key aspect of wellbeing, emphasising the importance of teams supporting each other and working together with compassion in building a health and wellbeing culture (NHS Employers, 2021), from this perspective the clinical and wellbeing lead (where in place) is pivotal in embodying culture change and modelling desirable behaviours (see recommendation 4).

Daniels J, *et al. Emerg Med J* 2024; 41:257–265. doi: 10.1136/emermed-2023-213189

b. Inter-professional valuing and respect

The dynamics in the ED are complex; roles have evolved over the previous 50 years and the hierarchy and traditional values continue to prevail in (some) EDs. More work needs to be done by RCEM to draw out the uniquely valuable aspects of each professional role, highlighting the necessary mixed skill set required in the delivery of optimum care and team functioning. Many PIPP staff reported feeling under-valued by their professional body and their team, particularly nursing staff, SAS doctors and ACP. The issue of tackling these traditional values and systems ties closely with the concept of workforce redesign; it is important to seek new ways of working and support staff to develop their skills to meet the needs of new organisation systems and new challenges in the current climate. Similarly, it is important that when these changes are made, staff are engaged in this process and are supported when adapting to these changes. As outlined in the CARES policy, staff must proactively address incivility within specialties, adhering to up-to-date policies in relation to bullying, harassment and issues of inclusion. This does however also extend to cross-specialty interactions (outside of ED) and the interface between trusts. Intergroup conflict can have a significant influence on the quality of patient care (Hewett et al. 2009). The development of clearer referral processes and the use of internal professional standards of what should occur in cases of disputed referral or other disagreements would safeguard against incivility emerging as a result of confused procedural matters such as referrals and caseload management.

c. Clarifying lines of accountability

Lines of accountability are reported to be unclear in the ED. While the clinical lead and matrons may assume key roles of responsibility, with wellbeing leads taking responsibility for initiatives related to team functioning and wellbeing, there has been lack of clarity in relation to raising issues and who is responsible, which has been partly attributed to a culture of blame. This has discouraged staff from raising issues; they are often perceived to be unwelcome or are not progressed. While change processes and managerial structures change from site to site, lack of understanding around these processes ultimately results in necessary changes not taking place, risking errors and patient safety. It is essential that the channels of communication between the ED and the wider trust are open and more visible to operational and non-clinical managers; staff on the 'shop floor' should have a good understand of how they can be agents of change in the system. This could take the form of 'safety huddles' for registrars in charge - safety huddles are short multidisciplinary briefings held at a particular time and place, focused on reducing risk in patients, celebrating success and learning from previous actions. These huddles can allow teams to improve communication and discuss additional topics such as role and training needs with more senior leaders (NHS England, 2019). They should be inclusive of all professional groups to ensure each perspective is accounted for. A clear written/visual description of the lines of accountability from shop floor clinician to chief executive should form part of trust and local induction material – it is by no means intuitive.

d. Nurturing growth

Many staff voiced concerns that their progress and wellbeing was not a priority to anyone, and that no one was particularly interested in their progress. Growth within the profession is again vital to job satisfaction, wellbeing and retention. This can be addressed through regular planned formal and informal discussions or reviews of educational needs, personal needs and circumstances, at a frequency acceptable to those involved. This should also form part of an appraisal that is tailored to the individual, completed with dedicated time, space and interest in the interviewee. The GMC's 'ABC' of core needs outlines that senior leaders should consult all clinical staff and gather feedback about how healthcare teams are established and work is organised, "focusing on learning and not blame" (GMC, 2019). For example, RCEM have provided consultant workforce recommendations to help support more sustainable careers (RCEM, 2018), including supporting less than full time working careers and development of individual job plans to allow for better balance of clinical and non-clinical work. Examples of best practice can also be found in the SAS development and retention programme (HEE, 2018). It is vital that each staff member feels that their personal growth is nurtured, an aspect of a well-

Daniels J, *et al. Emerg Med J* 2024; 41:257–265. doi: 10.1136/emermed-2023-213189

functioning workplace culture (Manley et al. 2019) and an investment in retaining the workforce; Radhakrishna (2015) also highlights the importance of training to retain staff when addressing cultural change.

Variety in role should also form part of reviewing progress and growth within the specialty; particularly for those in roles where they may be allocated a particular section, such as nurses, or who have been working in the same ED for an extended period or nearing retirement. This could include short-term or long-term secondments, or day-to-day rotations. This is in line with the RCEM CARES policy relating to retention, however this needs to be implemented at a local trust level, with processes in place to be able to request variation; this should fall within the remit of clinical leadership. Considerable focus has been given to workforce redesign as a strategy for developing the workforce, retaining staff and improving patient care (NHS Employers, 2021). Workforce re-design can range from designing new roles for staff based on skill sets; developing new models to allow staff to work in other areas of expertise; and increasing opportunities for skill development such as apprenticeships. As part of the NHS Long Term Plan, there is a focus on developing integrated care systems by working with clinical staff to develop more generalist skills and broaden their expertise to allow them to work across organisational boundaries and work more effectively to meet patient needs (HEE, 2022); this model of working would allow a broader scope for continuing professional development.

e. *Team cohesion*

Staff highlighted how well teams can and do work together in the ED, and how interdependent they are as a multidisciplinary team. Staff should be supported to develop their team cohesion through regular team activities, team training, and opportunities to receive structured and bespoke support as a team (see recommendation 3).

Staff collaboration and team cohesiveness has been highlighted as a key area central to positive culture change and a core value found in effective workplaces (Manley et al, 2019), can positively impact on patient care (Weaver et al. 2014) and can protect from negative impacts from providing day-to-day patient care (Maben et al. 2012). The Royal College of Physicians (RCP) have previously published resources to help improve team working in healthcare, including identifying the key agents of change. The resources are split into four key components: building effective teams, team culture, team communication and team development (RCP, 2017). Due to the high-turnover, high numbers of staff on rota and the 24 hour nature of the ED, specific attention much be directed toward addressing issues of team cohesion, this might relate to an overarching wellbeing strategy that encompasses group cohesion, however this must be conscious of the nature barriers that ED working presents, such as staff turn-over, high stress and workload, and lack of incentive.

Recommendation 3: A tailored pathway of care from ED to staff support

A tailored pathway of care is vital for staff to access the care they need when they need it. As highlighted previously in the CoCCo study (Daniels et al. 2021), ordinary working conditions for ED staff (e.g. shift patterns, high work stress, high workload and irregular hours) naturally present barriers to accessing care. In our previous work we outlined the necessity of a coherent pathway tailored to need, however staff support provision continues to vary by trust as there is little in place to support implementation. This may be partly attributable to a lack of consensus around how a staff support service modelled and the lack of evidence relating to a particular approach.

To support the implementation of a tailored care pathway from ED to evidence-based staff support, two award winning staff support services (Alderhay Children's Hospital Staff Advice and Liaison Service (SALS) model; North Bristol NHS Trust Staff Support Service) and psychological services as PIPP pilot project sites were consulted in order to co-create an exemplar model of staff support which was a) underpinned by work package 1 empirical data identifying needs and preferences b) aligned to current

guidance, including NICE (2022) guidance on mental wellbeing at work and consistent with recommendation 31 of the HEE mental wellbeing commission report, the NHS People Promise (NHS England, 2020)/NHS wellbeing framework (NHS Employers, 2021) and c) shaped by experts in the field (JP and OD) (see figure 1).

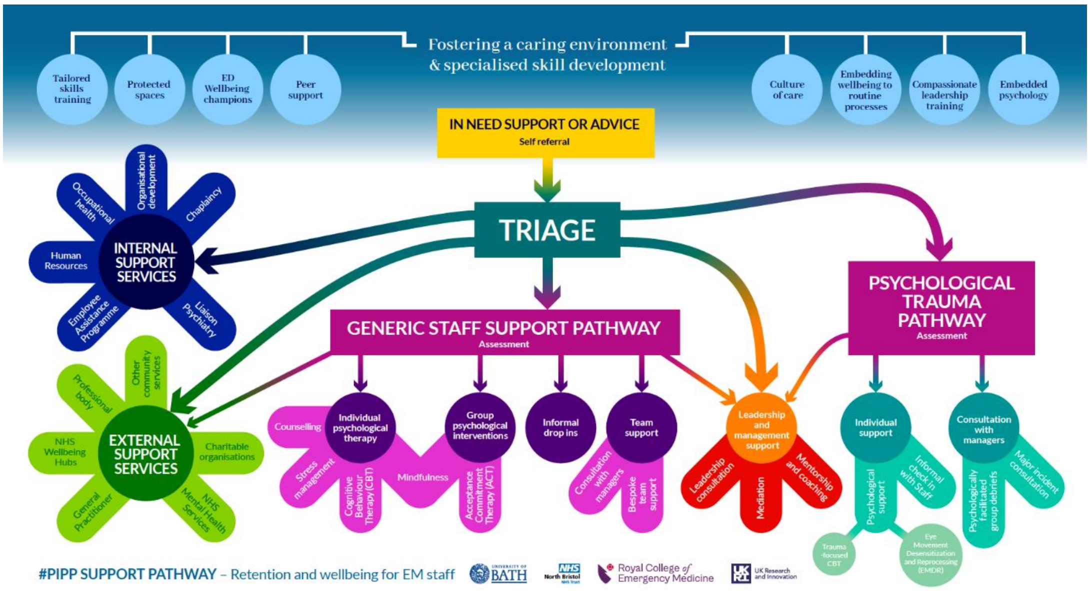

Figure 1. Exemplar model of staff support

Echoing findings from the CoCCo study, data from work package 1 supports a multi-level approach which is tailored to need, offers choice, and is clearly and easily accessible to all. From consulting both experts, and through a review of other wellbeing services across the UK, a shift was noted towards 'staff support' rather than 'psychological wellbeing' services, reflecting that these services are not developed exclusively for those struggling with mental health, but for staff who are in need of 'support'. In summary, these exemplar models of staff support include:

a) proactive/preventative measures to create a positive high-functioning team that are well equipped to manage challenges. This includes a background culture of care and shared responsibility for psychological wellbeing. The RCN defines a healthy workplace as one which takes a 'proactive approach to tackling the work-related factors that can lead to stress and poor mental health and building on established evidence on what constitutes good work' (RCN, 2021[2]). This is reflected in the top blue banner of figure 1.

b) interventions that are responsive to need, from bespoke team training to leadership consultation, individual structured therapy to informal drop-ins. As reflected in 1, there are two pathways into the service (generic, trauma related) and then three further streams relating to the recipient of the intervention (individual, team, leadership) which offer different format and modes of evidence-based support and interventions, if staff are not signposted out to a more

appropriate service. It has been emphasised that are interventions and interactions are ‘trauma informed’, which is of particular relevance to ED clinical staff.

A more detailed description of this pathway of care is available in appendix A of the main PiPP report. What is key from the empirical data is that staff support is provided locally, by staff who are known to the team, and importantly, that the culture dictates wellbeing is prioritised by all, for all. This includes regular informal wellbeing ‘check ins’ with wellbeing firmly on the formal appraisal agenda; the International Public Policy Observatory (IPPO) recommends prioritising the mental health and safety of staff, suggesting that organisations should use operationally focused tools to monitor the wellbeing of staff on a regular basis (IPPO, 2022). However, this support is redundant if staff are not supported to access the service; protected time and a culture recognising the impact of intensive ED working is necessary to facilitate access to support when needed.

The appointed wellbeing lead in the ED plays a pivotal gatekeeper role in accessing staff wellbeing support and attitudes towards wellbeing in the ED. The wellbeing lead role should be clearly defined, and should include the following:

- Role-model positive and healthy behaviours consistent with a culture of care, including adhering to breaks and annual leave, self-care, normalising psychological responses to difficult experiences
- Provide visible, regularly renewed information on staff support services and wellbeing matters
- Advise on available support services (including those signposted to in figure 1) and methods of access
- Deliver ‘warm handover’ – ensure that those seeking help to reach the required support
- Innovate initiatives to support staff wellbeing, e.g. staff team activities, informal check ins, wellbeing surveys (which are then acted upon).

Recommendation 4: Enhanced Leadership

Leadership is key to all areas within these recommendations: leaders are the key agents of change in the ED and are critical to the successful functioning of teams and individuals, this includes clinical leads, matrons and non-clinical management. However, many are unsupported across a number of domains, including delivery of responsibility or tasks they do not feel skilled to do. Leadership of teams is a key focus of the CQC when evaluating healthcare organisations; leaders are pivotal in promoting an open, caring culture as well as supporting learning and innovation. According to the CQC, leaders should strive to be compassionate, inclusive and approachable (CQC, 2018). The Institute for Healthcare Improvement (IHI) view leadership as essential in driving change in culture and organisational transformation, therefore investment is a crucial component in a drive to address retention in the ED. The role and quality of leadership is also a key influence in all PIPP domains of recommendation.

RCEM provides training in the form of the 'EM Leaders' framework and through Communities of Practice memberships. Both offer leadership training and e-learning courses, that help to up-skill those currently working in EDs, however there is increasing focus toward 'compassionate leadership' with King' Fund placing heavy emphasis on this approach. Compassionate leadership training is commonly provided by staff support services, as depicted in figure 1.

Key steps in enhancing leadership

Those in leadership positions should be supported to:

- Access to compassionate, inclusive leadership training and protected time to do so
- Engage with a leadership mentorship or coaching programme to support continued development in the role and create safe spaces to problem-solve and reflect
- Access to consultation with the wellbeing team as and when necessary (see figure 1).
- Gain a clear understanding of their roles through a defined role description, developed and provided by RCEM, to be used by staff to negotiate role responsibilities at trust level. Role descriptions should include protected additional time to undertake the additional responsibilities associated with a clinical lead role
- Consultants can engage with the clinical lead network, which should be broadened to include a platform or virtual environment where resources can be shared and accessed (e.g. a repository)
- Provide proactive, rather than reactive problem solving, with confidence, support and knowledge to escalate concerns when necessary.
- Recognise the pivotal role they play through role-modelling work positive behaviours such as taking breaks, normalising experiences, destigmatising distress, and openness to learning and change.
- As per NICE guidance and RCEM EmPower campaign, those in leadership positions should also be supported to attend leadership training as part of their workplan, and within their workplace hours as a necessary workplace activity (i.e. protected time). This also includes any follow-up training, mentorship or assignments. Following from this training, barriers to implementing this training into practice should also be addressed.

Project led by Dr Jo Daniels, University of Bath, UK. This work represents a collaboration between the Royal College of Emergency Medicine and University of Bath, funded by the UKRI. Full report available from j.daniels@bath.ac.uk, other documentation also available here: **Workforce | RCEM**
